# Octopamine Underlies the Counter-Regulatory Response to a Glucose Deficit in Honeybees (*Apis mellifera*)

**DOI:** 10.3389/fnsys.2017.00063

**Published:** 2017-08-30

**Authors:** Christina Buckemüller, Oliver Siehler, Josefine Göbel, Richard Zeumer, Anja Ölschläger, Dorothea Eisenhardt

**Affiliations:** Neurobiologie, Institut für Biologie, Fachbereich Biologie, Chemie, Pharmazie, Freie Universität Berlin Berlin, Germany

**Keywords:** honeybee, octopamine, glucose deficit, feeding state, hunger, hemolymph, survival, PER

## Abstract

An animal’s internal state is a critical parameter required for adaptation to a given environment. An important aspect of an animal’s internal state is the energy state that is adjusted to the needs of an animal by energy homeostasis. Glucose is one essential source of energy, especially for the brain. A shortage of glucose therefore triggers a complex response to restore the animal’s glucose supply. This counter-regulatory response to a glucose deficit includes metabolic responses like the mobilization of glucose from internal glucose stores and behavioral responses like increased foraging and a rapid intake of food. In mammals, the catecholamines adrenalin and noradrenalin take part in mediating these counter-regulatory responses to a glucose deficit. One candidate molecule that might play a role in these processes in insects is octopamine (OA). It is an invertebrate biogenic amine and has been suggested to derive from an ancestral pathway shared with adrenalin and noradrenalin. Thus, it could be hypothesized that OA plays a role in the insect’s counter-regulatory response to a glucose deficit. Here we tested this hypothesis in the honeybee (*Apis mellifera*), an insect that, as an adult, mainly feeds on carbohydrates and uses these as its main source of energy. We investigated alterations of the hemolymph glucose concentration, survival, and feeding behavior after starvation and examined the impact of OA on these processes in pharmacological experiments. We demonstrate an involvement of OA in these three processes in honeybees and conclude there is an involvement of OA in regulating a bee’s metabolic, physiological, and behavioral response following a phase of prolonged glucose deficit. Thus, OA in honeybees acts similarly to adrenalin and noradrenalin in mammals in regulating an animal’s counter-regulatory response.

## Introduction

An animal’s internal state is a critical parameter required for efficient decision-making toward a behavior that satisfies the animal’s needs in a given environment (Rangel et al., 2008). An important aspect of an animal’s internal state is the energy state that is adjusted to the needs of an animal by energy homeostasis. Glucose is an essential source of energy, especially for the brain, and a shortage of glucose therefore triggers a complex response to restore the animal’s glucose supply. In mammals, this includes metabolic responses like the mobilization of glucose from internal glucose stores in order to guarantee a constant glucose supply for the brain but also behavioral responses like foraging and a rapid food intake (Ritter et al., 2011). Glucose metabolism in mammals is regulated by the autonomic nervous system, consisting of the parasympathetic and the sympathetic nervous system that together orchestrate the interplay between different metabolic organs. The sympathetic nervous system connects to its target organs via noradrenalin and adrenalin. During a glucose-deficit sympathetic activity increases hepatic glucose output, stimulates glucagon release from the pancreas, inhibits pancreatic insulin release, and blocks glucose uptake in skeletal muscles (reviewed in Nonogaki, 2000; Verberne et al., 2014, 2016; Seoane-Collazo et al., 2015; Elliott et al., 2016). Furthermore, central noradrenergic neurons are involved in behavioral responses to a glucose deficit (Ritter et al., 2001, 2011; Li et al., 2014).

Octopamine (OA), an invertebrate biogenic amine, is similar to adrenalin and noradrenalin in its synthesis, its synthesizing enzymes, and the respective receptors; it is therefore suggested that adrenalin, noradrenalin, and OA derive from one ancestral pathway (Gallo et al., 2016). Based on OA’s involvement in the fight-or-flight response, motivation, and aggression and it’s adipokinetic function in insects a similarity of function between OA in insects and the biogenic amines adrenalin and noradrenalin in vertebrates has been suggested (reviewed in Roeder, 2005). Interestingly, in fruit flies, OA plays a role in starvation-induced hyperactivity (Yang et al., 2015; Yu et al., 2016) and regulates insulin-release, hemolymph sugar concentration (Li et al., 2016), and feeding behavior (Zhang et al., 2013). It can therefore be hypothesized that the mechanisms that regulate an animal’s response to a glucose deficit might be evolutionary conserved.

We here tested the hypothesis that the response to a glucose deficit is evolutionary conserved in honeybees (*Apis mellifera*). Adult forager bees feed mainly on carbohydrates and use carbohydrates as their main source of energy, but have no substantial carbohydrate, protein, or lipid reserves and only low glycogen stores in their bodies (Blatt and Roces, 2001; Hrassnigg and Crailsheim, 2005; Ihle et al., 2014; Paoli et al., 2014). Accordingly, a tight control of their sugar metabolism as well as their feeding behavior is necessary to avoid starvation. Therefore, we hypothesized that bees would show a counter-regulatory response to a glucose deficit that might be regulated by OA. We here tested this hypothesis and investigated alterations of the hemolymph glucose concentration, survival, and feeding behavior after starvation in pharmacological experiments.

We demonstrated an involvement of OA in regulating the hemolymph glucose concentration, survival, and feeding behavior. Thus, OA in honeybees acts similarly to adrenalin and noradrenalin in mammals in regulating an animal’s counter-regulatory response to a glucose deficit.

## Materials and Methods

### General Treatment of Honeybees

Forager bees from the garden of the Neurobiology Institute, Freie Universität Berlin, Germany were caught 1 day before the experiment, cooled on ice until immobilization and harnessed in plastic tubes. In the evening, around 4:00 p.m., bees were fed to satiation with 30% (w/v) sucrose solution (0.88 M). Overnight they were placed in a dark and humid box at room temperature. On the following day, experiments started at 10:00 a.m. (Felsenberg et al., 2011). When experiments took longer than 24 h, bees were fed each subsequent day at 4:00 p.m. four drops (4 μl each) of 30% (w/v) sucrose solution (0.88 M in tap water) unless otherwise noted.

### Drug Injection

Drugs were injected into the flight muscle as has been demonstrated in Felsenberg et al. (2011). A small hole was made in the cuticle above the flight muscle with a hypodermic needle (Sterican, G21, Braun, Melsungen), and with a glass capillary tube (Selzer, Labortechnik, Waghäusel) 1 μl of the solution was injected through the hole into the flight muscle.

### Measurement of Hemolymph Glucose Concentration

Following the protocol of Rether (2012), hemolymph (1–2 μl) was collected 15 min following drug injection with a microliter syringe (Hamilton) and a hypodermic needle (Sterican G30, Braun) on the lateral abdomen between two (4th and 5th) tergites. The hemolymph was applied to blood glucose test stripes (Accu-Chek Aviva, Roche Diabetes Care) and the glucose-concentration was measured with a blood sugar meter (Accu-Chek Aviva, Roche Diabetes Care).

### Survival of Honeybees

Honeybees were caught, harnessed, and fed as indicated above. Two experiments were carried out. Both experiments started the day after capture: 18 h after the bees had been fed to satiation they were divided into three subgroups that were systemically injected in the flight muscle with 1 μl OA (10 mM), epinastine (40 mM), or PBS (137 mM NaCl, 2.7 mM KCl, 10.1 mM Na_2_HPO_4_, 1.8 mM KH_2_PO_4_). Bees in the two experiments were treated differently following drug injection. In the first experiment bees remained unfed following drug injection until they died. In the second experiment bees were fed with 30% (w/v) sucrose solution to satiation 2 h following drug injection and were left unfed subsequently until they died. In both experiments, bees were inspected every 6 h after injection and survival was noted. The survival score for each bee was calculated from the number of time points the bee was still alive.

### Proboscis Extension Response

The proboscis extension response (PER) was released with three solutions: water (H_2_O), 0.1% (w/v) sucrose solution, i.e., 2.9 mM sucrose, and 43% (w/v) sucrose solution, i.e., 1.25 mM sucrose. The bees’ antennae were touched with a toothpick covered with one of these solutions and the extension of the bees’ proboscises was noted. Solutions were presented in an ascending order [first water, second 0.1% (w/v) sucrose solution, third 43% (w/v) sucrose solution] after intervals of 2 min.

### Statistics

Statistics were carried out with Prism 6.0 (Graph Pad) and Statistica 10.0 (Statsoft).

### Ethics Statement

This study involved insects, i.e., honeybees (*Apis mellifera*). The study was carried out in accordance to the Deutsche Tierschutzgesetz.

## Results

### Octopamine Increases the Hemolymph Glucose Concentration Depending on the Feeding State

First, we tested the hypothesis that OA is involved in the response to a glucose deficit in honeybees. Therefore, we examined whether OA is involved in regulating the honeybee’s hemolymph glucose level depending on its feeding state.

**FIGURE 1:**
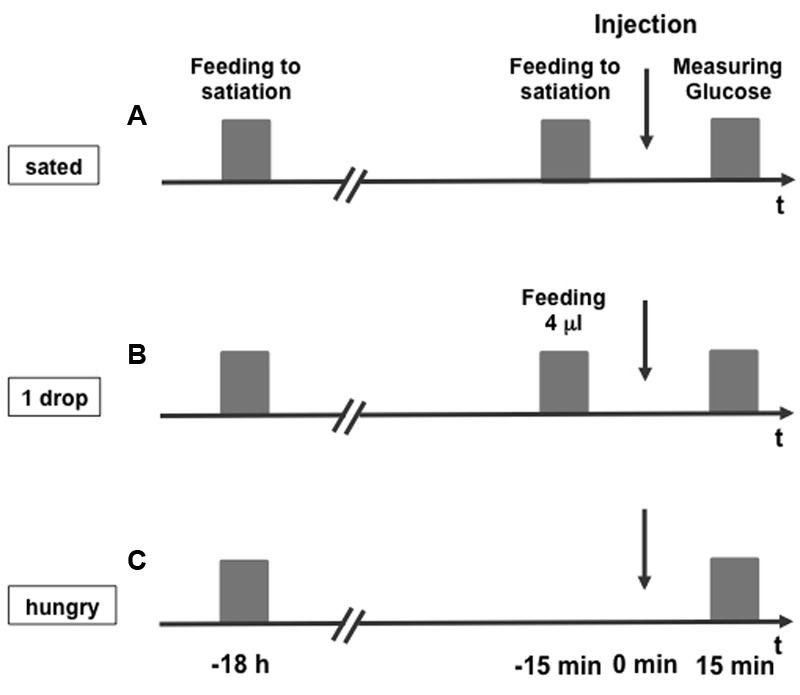
Experimental scheme for measuring the hemolymph glucose concentration of honeybees. Honeybees were fed to satiation (**A**, sated), fed with 4 μl 30% (w/v) sucrose solution (0.88 M) (**B**, 1 drop), or remained unfed (**C**, hungry) 15 min before they were systemically injected with octopamine, epinastine, or PBS (injection). Fifteen minutes following the injection hemolymph glucose concentration was measured.

We analyzed three groups of bees: Bees that were fed with 30% (w/v) sucrose solution to satiation, i.e., until they did not extend the proboscis anymore, 15 min before probing the hemolymph glucose level, bees fed with 4 μl of 30% (w/v) sucrose solution, and bees that were not fed at the same time point (**Figure 1**).

**FIGURE 2:**
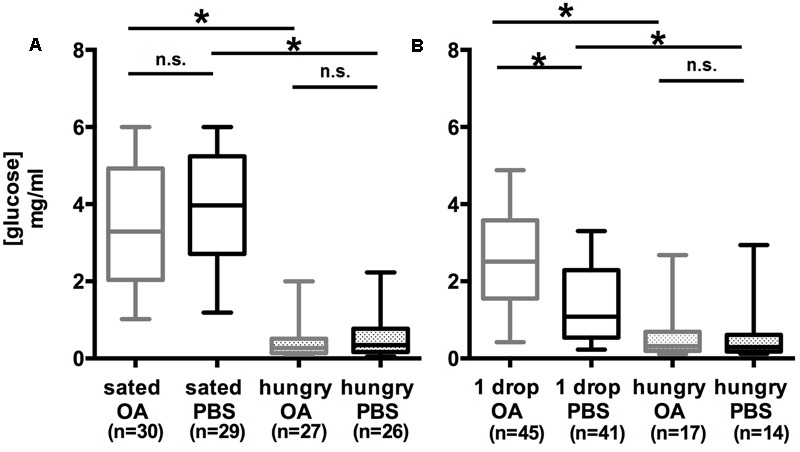
High hemolymph glucose concentration in honeybees following octopamine treatment depends on the feeding state. **(A)** The hemolymph glucose level of bees fed with 30% (w/v) sucrose solution (0.88 M) to satiation (sated PBS) was higher than that of hungry bees that remained unfed (hungry PBS). Octopamine had no effect on the hemolymph glucose concentration of sated (sated PBS vs. sated OA) and hungry (hungry PBS vs. hungry OA) bees. **(B)** The glucose level in the hemolymph of bees fed with 4 μl 30% (w/v) sucrose solution (0.88 M) (1 drop PBS) was higher than that of hungry bees (hungry PBS). Octopamine increased the hemolymph glucose concentration of bees fed with 4 μl 30% (w/v) sucrose solution (0.88 M) (1 drop PBS vs. 1 drop OA). ^∗^*p* < 0.05. Number of bees appears in brackets.

In two experiments, we tested the impact of OA on the hemolymph glucose level (**Figure 2**). In the first experiment (**Figure 2A**), honeybees that were fed to satiation (sated), and honeybees, that were not fed at the same time point (hungry) were compared. In the second experiment (**Figure 2B**), honeybees that were fed with 4 μl of 30% (w/v) sucrose solution (1 drop), were compared with honeybees that remained unfed (hungry). Each of the two groups was divided into two subgroups that were systemically injected with either 10 mM OA solved in PBS or with PBS alone. Fifteen minutes later 1–2 μl of hemolymph were taken from the bees’ abdomen, applied to a blood glucose test strip and measured with a blood glucose meter.

Comparison of the PBS-injected groups demonstrated that the hemolymph glucose concentration of hungry bees was significantly lower than the glucose concentration in sated bees was [**Figure 2A**; Kruskal–Wallis test: *H*(2, *N* = 82) = 54.99; PBS_sated_/PBS_hungry_: *p* = 8.0 E-9] and in bees fed with 4 μl sucrose (1 drop) [**Figure 2B**; Kruskal–Wallis test: *H*(2, *N* = 72) = 18.52; PBS_1drop_/PBS_hungry_: *p* = 0.0012].

The hemolymph glucose concentration of bees fed with 4 μl 30% (w/v) sucrose solution (1 drop) was significantly higher in bees injected with OA than in bees injected with PBS [**Figure 2B**; Kruskal–Wallis test: *H*(2, *N* = 103) = 33.64; OA_1drop_ vs. PBS_1drop_: *p* = 0.00054].

In both experiments, no significant differences between the hemolymph glucose levels of hungry bees that were injected with OA or PBS were observed [**Figure 2A**; Kruskal–Wallis test: *H*(2, *N* = 72) = 18.52; OA_hungry_ vs. PBS_hungry_: *p* = 1; **Figure 2B**; Kruskal–Wallis test: *H*(2, *N* = 82) = 54.99; OA_hungry_ vs. PBS_hungry_: *p* = 1]. The same holds true for the hemolymph glucose levels of the sated bees [**Figure 2A**; Kruskal–Wallis test: *H*(2, *N* = 86) = 54.19; OA_sated_ vs. PBS_sated_ = 1].

Taken together, these experiments demonstrated an enhancement of the hemolymph glucose level by OA in bees that were fed with a small amount of sucrose, whereas in hungry and in sated bees the effect of OA was not observed.

### Epinastine Inhibits the Hemolymph Glucose Concentration Depending on the Feeding State

**FIGURE 3:**
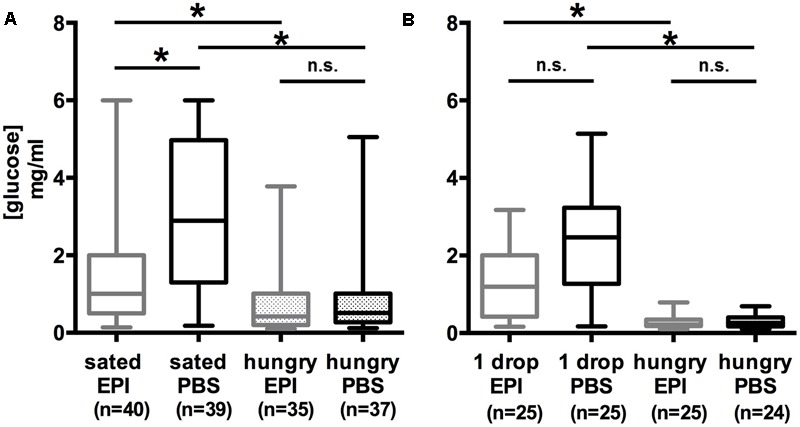
Low hemolymph glucose concentration in honeybees following epinastine treatment depends on the feeding state. **(A)** The hemolymph glucose level of bees fed with 30% (w/v) sucrose solution (0.88 M) to satiation (sated PBS) was higher than that of hungry bees that remained unfed (hungry PBS). Sated and epinastine-treated bees showed a lower hemolymph glucose concentration than sated control animals (sated EPI vs. sated PBS). Epinastine-treatment did not affect hungry bees (hungry EPI vs. hungry PBS). **(B)** The glucose level in the hemolymph of bees fed with 4 μl 30% (w/v) sucrose solution (0.88 M) (1 drop PBS) was higher than that of hungry bees (hungry PBS). Epinastine-treatment did not affect bees fed with 4 μl 30% (w/v) sucrose solution (0.88 M) (1 drop EPI vs. 1 drop PBS) and hungry bees (hungry EPI vs. hungry PBS). ^∗^*p* < 0.05. Number of bees appears in brackets.

In order to verify our finding of an effect of OA on the feeding-dependent hemolymph glucose concentration we next examined the effect of epinastine (EPI), an OA-receptor antagonist (Roeder et al., 1998), in the three groups of differently fed bees, i.e., bees that remained hungry, bees that were fed 4 μl of 30% (w/v) sucrose solution, and bees that were fed to satiation. Again, two different groups of bees were compared in two experiments, sated vs. hungry bees (**Figure 3A**) and hungry bees vs. bees that were fed with 4 μl of 30% (w/v) sucrose solution (1 drop) (**Figure 3B**). In these experiments, 40 mM EPI dissolved in PBS or PBS alone were injected 15 min following feeding and 15 min before probing the hemolymph glucose level.

Comparing the PBS-injected groups revealed a significantly lower concentration of hemolymph glucose in bees that remained unfed (hungry) compared to sated bees [**Figure 3A**; Kruskal–Wallis test: *H*(2, *N* = 111) = 39.43; PBS_hungry_/PBS_sated_: *p* = 1.6 E-6] and hungry bees compared to bees fed with 4 μl of 30% (w/v) sucrose solution (1 drop) [**Figure 3B**; Kruskal–Wallis test: *H*(2, *N* = 74) = 42.91; PBS_1drop_/PBS_sated_: *p* = 1.7 E-7].

In sated bees that received an EPI injection the hemolymph glucose concentration was significantly lower than the glucose concentration of bees injected with PBS [**Figure 3A**; Kruskal–Wallis test: *H*(2, *N* = 114) = 34.40; EPI_sated_/PBS_sated_: *p* = 0.0049], whereas in bees fed with 4 μl sucrose (1 drop) the difference in hemolymph glucose concentration after EPI- and PBS-injection was not significant [**Figure 3B**; Kruskal–Wallis test: *H*(2, *N* = 75) = 39.56; EPI_1drop_/PBS_1drop_: *p* = 0.065], but the results suggested a less pronounced increase in glucose concentration in bees injected with EPI.

The difference in hemolymph glucose between hungry bees injected with EPI and PBS was not significant [**Figure 3A**; Kruskal–Wallis test: *H*(2, *N* = 111) = 39.43; EPI_hungry_/PBS_hungry_: *p* = 1; **Figure 3B**; *H*(2, *N* = 74) = 42.91; EPI_hungry_/PBS_hungry_: *p* = 1].

Taken together, these experiments demonstrated an inhibitory effect of EPI on the hemolymph glucose level in sated bees but not in bees that were fed with 4 μl sucrose solution and in hungry bees.

### Octopamine Decreases the Survival Rate of Hungry Bees

An appropriate energy supply is essential to maintain an animal during phases with an increased energy demand, for example, when the supply of nutrients is interrupted. Since foragers require a diet high in carbohydrates for survival and glucose is one of the main sugars found in the honeybee’s hemolymph (Beutler, 1936; Blatt and Roces, 2001; Ihle et al., 2014; Paoli et al., 2014) we next tested whether OA impacts survival of hungry bees.

**FIGURE 4:**
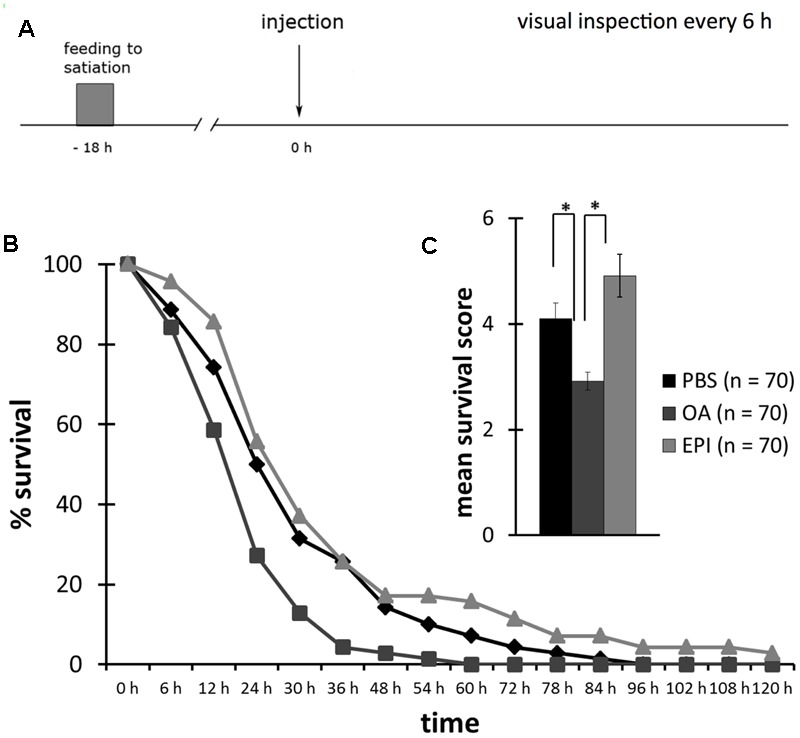
Reduced survival following octopamine-treatment of hungry honeybees. **(A)** Schematic overview of the experiment. Bees were fed to satiation with 30% (w/v) sucrose solution (0.88 M) 18 h before injection of octopamine (OA), epinastine (EPI), or PBS. Their survival was observed every 6 h. **(B)** Survival of bees injected with PBS (rhomb, black), OA (square, dark gray), or EPI (triangle, light gray). **(C)** Bees injected with OA had a significant lower survival score than bees injected with PBS or EPI. ^∗^*p* < 0.05. Number of bees appears in brackets.

We injected bees that were not fed for 18 h (hungry), with 10 mM OA, 40 mM EPI or PBS. We counted the bees that survived without food 16 times, i.e., every 6 h, until all bees were dead (**Figure 4A**).

The survival of bees injected with OA was significantly lower than of bees injected with EPI (**Figure 4B**, rm ANOVA, factor injection: *F*_22;394_ = 1.7447; *p* = 0.0206; Fisher LSD *post hoc* test: *p* = 0.0276). There was no difference between the survival of bees injected with OA or PBS (Fisher LSD *post hoc* test: *p* = 0.4064) and between bees injected with PBS or EPI (Fisher LSD *post hoc* test: *p* = 0.1671).

The mean survival score of OA-injected bees was significantly lower than of PBS- and EPI-injected bees (**Figure 4C**, one factor ANOVA, factor injection: *F*_2;207_ = 10.8305; *p* < 0.001; Fisher LSD *post hoc* test: PBS vs. OA: *p* = 0.0066; EPI vs. OA: *p* < 0.001). Bees injected with EPI had a higher survival score than did PBS-injected bees, but this difference was not significant (Fisher LSD *post hoc* test: *p* = 0.0610).

Taken together, this experiment demonstrated that OA decreased the survival rate of hungry bees and thus the time span they survive without food.

### Feeding Restores the Octopamine-Effect on the Bees Survival

**FIGURE 5:**
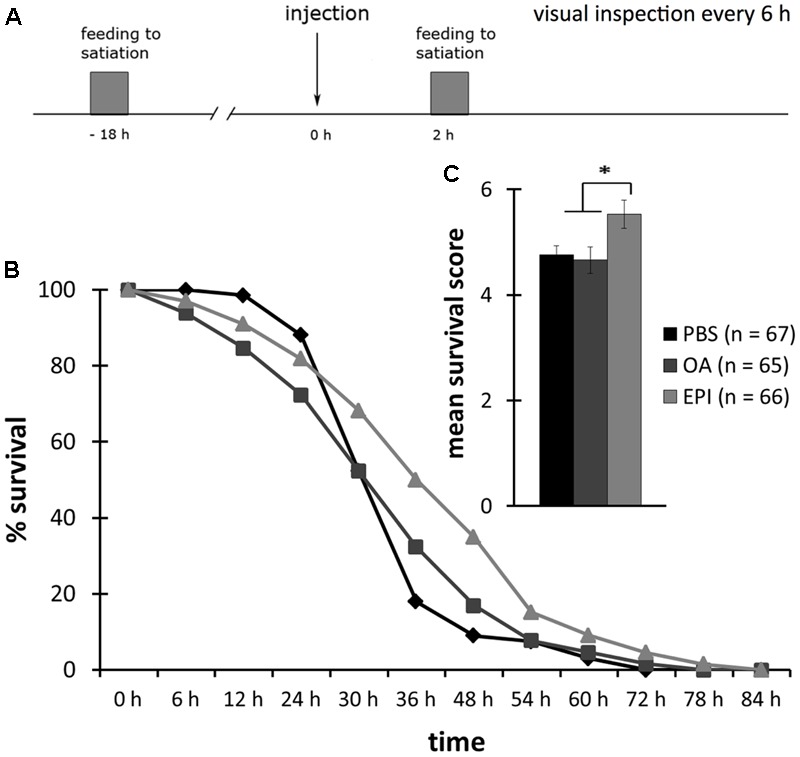
High survival rate of epinastine-treated honeybees that were fed to satiation. **(A)** Schematic overview of the experiment. Bees were fed to satiation with 30% (w/v) sucrose solution 18 h before injection of octopamine (OA), epinastine (EPI), or PBS. Two hours following drug injection, bees were again fed to satiation with 30% (w/v) sucrose solution (0.88 M). Their survival was observed every 6 h. **(B)** Survival of bees injected with PBS (rhomb, black), OA (square, dark gray), or EPI (triangle, light gray). **(C)** No difference in the survival score between bees injected with OA and PBS. EPI-treatment results in a higher survival score than PBS or OA treatment. ^∗^*p* < 0.05. Number of bees appears in brackets.

In a second experiment, we considered whether feeding of bees with sucrose following the OA- or EPI-injection restores survival. We again injected bees that were not fed for 18 h (hungry), with 10 mM OA, 40 mM EPI or PBS and fed them to satiation with 30% (w/v) sucrose solution 2 h following drug injection. We counted the number of bees that survived without food 12 times, i.e., every 6 h, until all bees were dead (**Figure 5A**).

The survival of bees injected with OA or PBS was significantly different (**Figure 5B**, rm ANOVA, factor injection: *F*_20;364_ = 2.0627; *p* = 0.0050; Fisher LSD *post hoc* test OA vs. PBS: *p* = 0.0403; OA vs. EPI: *p* = 0.3070; PBS vs. EPI: *p* = 0.3032). The survival rate of EPI-treated bees was higher compared to both PBS- and OA-treated bees (one-factor ANOVA, factor injection: *F*_2;195_ = 4.118; *p* = 0.0177; Fisher LSD *post hoc* test EPI vs. PBS: *p* = 0.0207; Fisher LSD *post hoc* test EPI vs. OA: *p* = 0.0096). OA- and PBS-injected bees showed no significant difference in their mean survival rates (**Figure 5C**, Fisher LSD *post hoc* test OA vs. PBS: *p* = 0.7637).

Taken together, this experiment demonstrated that feeding of sucrose 2 h following drug injection restored the OA-dependent decrease of the bees’ survival rate, and thus the time span of survival to the level of the PBS-injected control group. Furthermore, feeding resulted in a higher survival rate of bees injected with EPI compared to the PBS-injected control group and thus in an enhancement of the time span of survival following an EPI-treatment.

### The Proboscis Extension Response Depends on the Honeybees Feeding State

**FIGURE 6:**
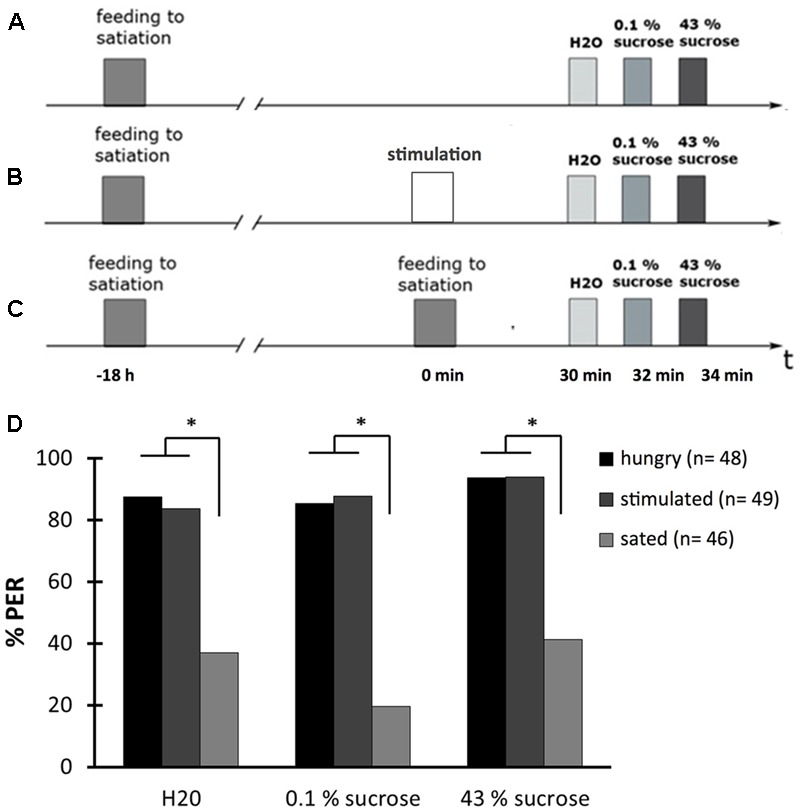
The proboscis extension response (PER) depends on the feeding state. **(A)** Schematic overview of the experiment. Bees were fed to satiation with 30% (w/v) sucrose solution (0.88 M) 18.5 h before testing the PER (hungry). The PER was tested with water (H_2_O), with 0.1% (w/v) sucrose solution (2.9 mM) (0.1% sucrose), and with 43% (w/v) sucrose solution (1.25 M) (43% sucrose). **(B)** Schematic overview of the experiment. Bees were fed to satiation with 30% (w/v) sucrose solution (0.88 M) 18.5 h before testing the PER and were stimulated at their antennae with 30% (w/v) sucrose solution (0.88 M) 30 min before testing the PER (stimulated). The PER was tested as described in **(A)**. **(C)** Schematic overview of the experiment. Bees were fed to satiation with 30% (w/v) sucrose solution (0.88 M) 18.5 h and again 30 min before testing the PER (sated). The PER was tested as described in **(A)**. **(D)** The PER depends on the feeding state of bees. The percentage of hungry bees responding with a PER is higher than the percentage of sated bees but the percentage of stimulated bees responding is as high as of hungry bees. ^∗^*p* < 0.05. Number of bees appears in brackets.

The response to a glucose deficit is characterized by metabolic and behavioral changes. Therefore, it was prudent to determine whether OA leads to an altered feeding behavior depending on the bees’ feeding state. Part of the feeding behavior of honeybees is the proboscis extension response (PER), which is a reflex-like response to a food stimulus: When the antennae or the proboscis of a honeybee are touched with sucrose solution, the bee extends its proboscis. However, when fed with sucrose, bees decrease this response until it is not elicited anymore. Above we demonstrated that bees that were fed with sucrose until extension could not be elicited anymore showed a higher hemolymph glucose concentration than did bees that were not fed or that were fed with 4 μl sucrose solution. Thus, it seemed likely that the feeding state impacts the PER. However, this is not entirely clear because multiple stimulations of the antennae with sucrose solution could lead to a decrease of the PER, i.e., habituation. We here tested the hypothesis that the feeding state impacts the PER and examined the PER in bees that were fed to satiation with 30% (w/v) sucrose solution 18.5 h before testing the PER. These bees were divided into three groups. One was not fed again before the PER test (hungry, **Figure 6A**), one group received multiple stimulations with 30% (w/v) sucrose solution to the antennae 30 min before the PER test (stimulated, **Figure 6B**), and one group that was fed again with 30% (w/v) sucrose solution to satiation 30 min before testing the PER (sated, **Figure 6C**). We tested the PER with water, 0.1% (w/v) sucrose solution, and 43% (w/v) sucrose solution 30 min after feeding, respectively stimulation of the antennae.

The percentage of bees responding with a PER was not different between the group that was not fed before the PER test and the group that received the sucrose stimulation (**Figure 6D**; rm ANOVA, factor treatment: *F*_2;140_ = 51.1503; *p* < 0.001; Fisher LSD *post hoc* test: hungry bees vs. stimulated bees: *p* = 0.9426). However, a statistically significant difference between these two groups and the fed bees was found: a lower percentage of fed bees responded to all three stimuli with a PER (**Figure 6D**; Fisher LSD *post hoc* test: sated bees vs. hungry bees: *p* < 0.001, stimulated bees vs. sated bees: *p* < 0.001).

Thus, the percentage of bees responding with a PER to water and sucrose stimulation depended on the feeding state of a bee and not on the repetitive stimulation of their antennae during feeding.

### OA-Injection Does Not Affect the PER in Hungry Bees or Sated Bees

**FIGURE 7:**
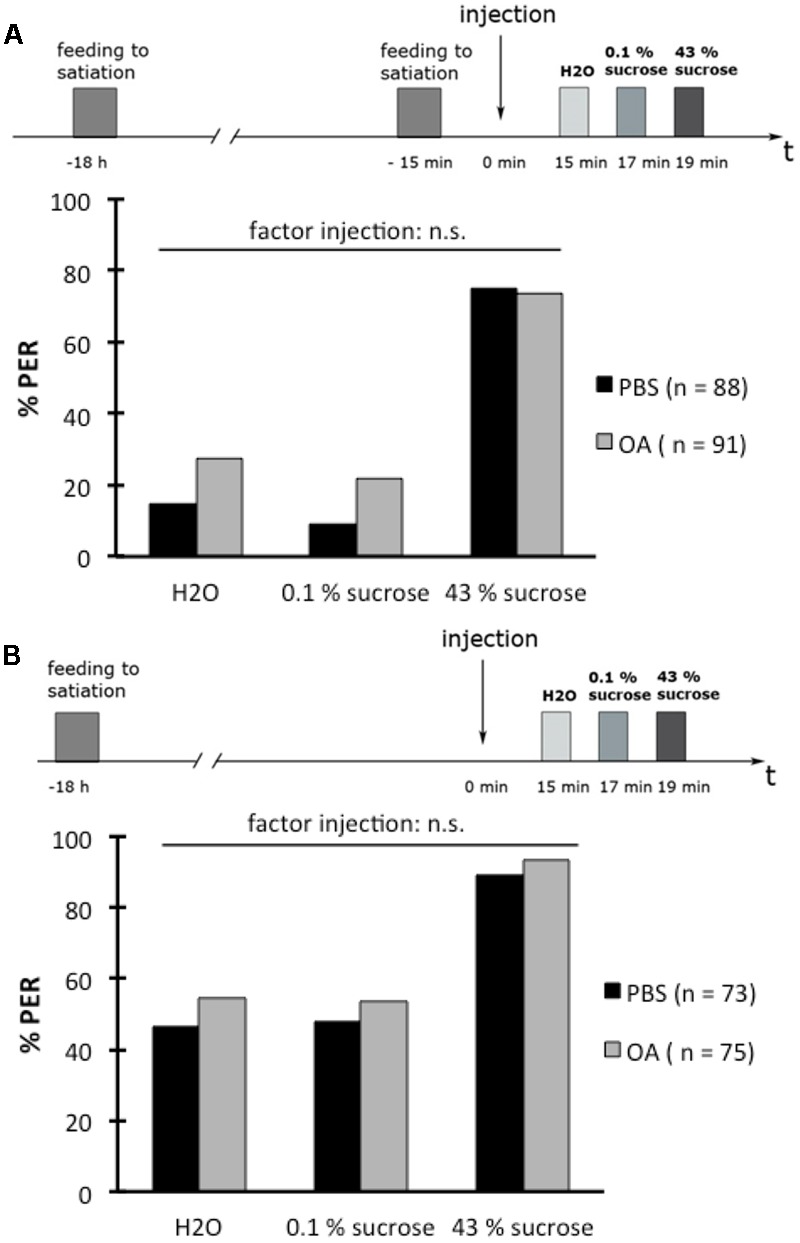
Octopamine does not affect the proboscis extension response (PER) of hungry or sated honeybees. **(A)** Schematic overview of the experiment (above). Bees were fed to satiation with 30% (w/v) sucrose solution (0.88 M) 18 h and again 15 min before drug injection. The PER was tested 15 min after drug injection with water (H_2_O), with 0.1% (w/v) sucrose solution (2.9 mM) (0.1% sucrose), and with 43% (w/v) sucrose solution (1.25 M) (43% sucrose). Octopamine (OA) did not affect the PER to these different solutions in sated bees (below). **(B)** Schematic overview of the experiment (above). Bees were fed to satiation with 30% (w/v) sucrose solution (0.88 M) 18 h before drug injection. The PER was tested 15 min after drug injection as described in **(A)**. OA did not affect the PER to these different solutions in hungry bees (below). Number of bees appears in brackets.

Next, we tested whether OA is involved in the PER depending on the bees’ feeding state. In two experiments that were done in parallel, we tested the PER with water, 0.1% (w/v) sucrose solution, and 43% (w/v) sucrose solution. In the first experiment, bees were examined that were fed with 30% (w/v) sucrose solution until the PER was not elicited anymore, 30 min before the PER test (sated) (**Figure 7A**). In the second experiment, bees that were not fed at the same time point (hungry) were tested (**Figure 7B**). In both experiments, bees were divided into two groups, those that received an injection of 10 mM OA or those that received PBS 15 min before the PER test.

OA-injection did not have an effect on the PER rate in both sated bees (**Figure 7A**; rm ANOVA, factor injection: *F*_1;177_ = 3.1551; *p* = 0.0774) and hungry bees (**Figure 7B**; rm ANOVA, factor injection: *F*_1;146_ = 1.1004; *p* = 0.2956) compared to the PER rate of bees injected with PBS.

### Epinastine Reduces the Proboscis Extension Response Rate in Hungry Bees

**FIGURE 8:**
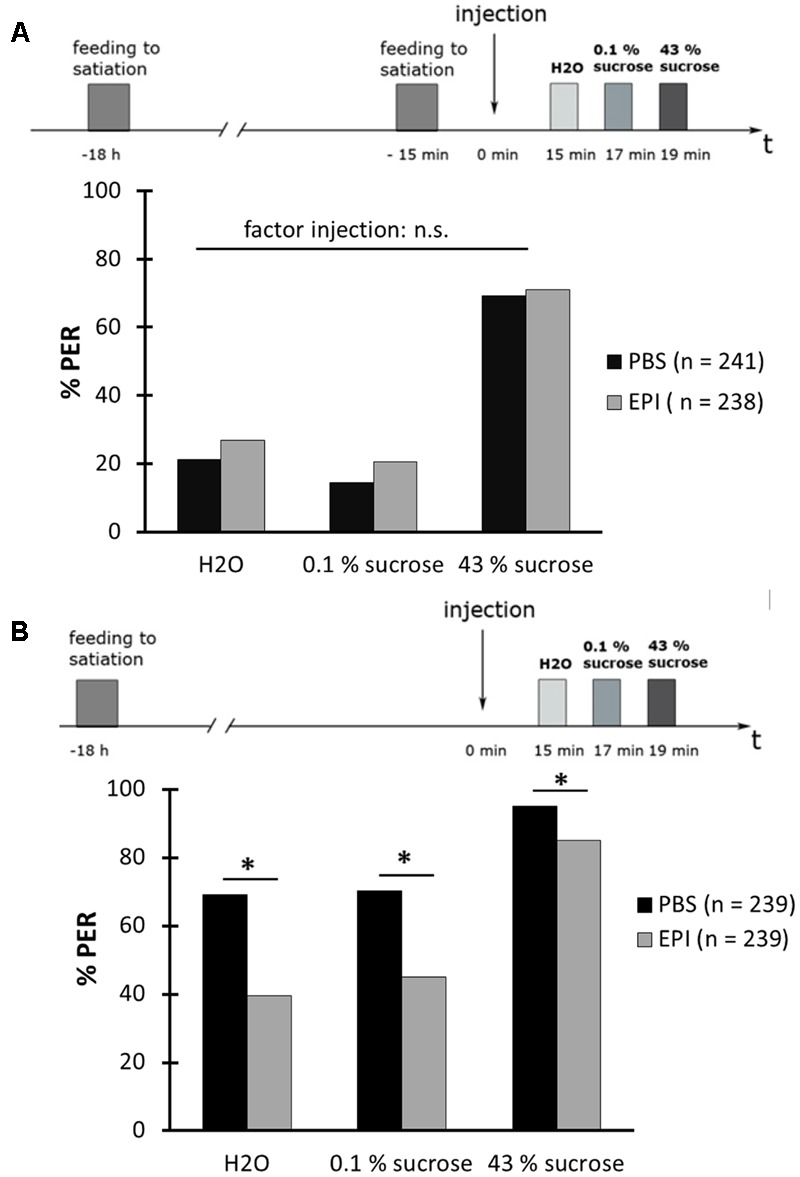
Epinastine blocks the proboscis extension response (PER) of hungry honeybees. **(A)** Schematic overview of the experiment (above). Bees were fed to satiation with 30% (w/v) sucrose solution (0.88 M)18 h and again 15 min before drug injection. The PER was tested 15 min after drug injection with water (H_2_O), with 0.1% (w/v) sucrose solution (2.9 mM) (0.1% sucrose), and with 43% (w/v) sucrose solution (1.25 M) (43% sucrose). Epinastine (EPI) did not affect the PER to these different solutions in sated bees (below). **(B)** Schematic overview of the experiment (above). Bees were fed to satiation with 30% (w/v) sucrose solution (0.88 M) 18 h before drug injection. The PER was tested 15 min after drug injection as described in **(A)**. EPI blocked the PER of hungry bees (below). ^∗^*p* < 0.05. Number of bees appears in brackets.

We next determined whether EPI affects the PER. Again, we carried out two experiments in parallel, one with bees that were fed to satiation 30 min before the PER-test (sated) (**Figure 8A**) and another with bees that were not fed at the same time point (hungry) (**Figure 8B**). Bees in both experiments were divided into two groups: one group that received an injection with EPI (40 mM) while the other group received PBS-injection. Fifteen minutes following these injections, the PER was tested successively with water, 0.1% (w/v) sucrose solution, and with 43% (w/v) sucrose solution.

In sated bees, the injection of EPI had no effect on the PER rate when compared to the PER rate of PBS-injected bees (**Figure 8A**; rm ANOVA, factor injection: *F*_1;477_ = 2.2498; *p* = 0.1343). Hungry bees showed a significantly lower PER rate after an injection with 40 mM EPI than after an injection with PBS (**Figure 8B**; rm ANOVA, factor injection × sugar solution: *F*_2;954_ = 12.843; *p* < 0.001).

This result suggested that EPI inhibits the high PER of hungry bees and that OA receptors, and thus OA, might be involved.

### α-Methyl-p-Tyrosine Inhibits the PER Rate in Hungry Bees and Can Be Rescued by Octopamine But Not Dopamine

Although we could not detect an effect of OA on the PER of honeybees the results of the previous experiment suggested that OA might be involved. Therefore, we next examined whether there is evidence that OA is required to modulate the PER in hungry bees. For this, we utilized the drug α-methyl-*p*-tyrosine (AMT). AMT inhibits synthesis of both OA and dopamine (DA) and therefore reduces the amount of biogenic amines (Stevenson et al., 2000). AMT does not block receptors irreversibly as is the case with the use of receptor antagonists. An injection of OA or DA can therefore restore the amount of these otherwise depleted amines. Accordingly, AMT was used to stop the synthesis of OA and DA to examine the effect of externally added OA and DA on the bees’ PER.

**FIGURE 9:**
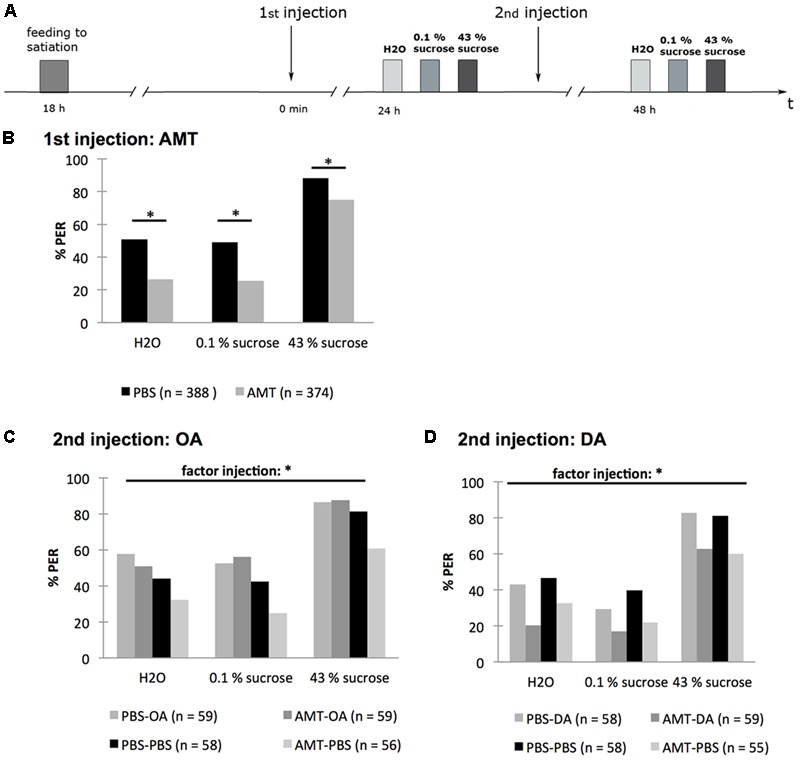
Octopamine but not dopamine rescues the effect of α-methyl-*p*-tyrosine (AMT) on the PER of honeybees. **(A)** Schematic overview of the experiment. Bees were fed to satiation with 30% (w/v) sucrose solution (0.88 M) 18 h before injection of AMT (first injection). Twenty-four hours following AMT injection the PER was tested touching the antennae with water (H_2_O), 0.1% (w/v) sucrose solution (2.9 mM) (0.1% sucrose), 43% (w/v) sucrose solution (1.25 M) (43% sucrose). Following this test, subgroups of bees were injected with octopamine (OA), dopamine (DA), or PBS. Another 24 h later the PER was tested again with water (H_2_O), 0.1% (w/v) sucrose solution (2.9 mM) (0.1% sucrose), 43% (w/v) sucrose solution (1.25 M) (43% sucrose). **(B)** One day after injection of AMT or PBS significant differences between groups were found. **(C)** Injection of OA rescued the AMT effect. **(D)** Injection of DA did not rescue the AMT effect. ^∗^*p* < 0.05. Number of bees appears in brackets.

In this experiment (**Figure 9A**), bees were fed to satiation 18 h before injection with 30.5 mM AMT. Twenty-four hours later the PER was tested with water, 0.1% (w/v) sucrose solution, and 43% (w/v) sucrose solution. Following this test, bees were injected with 10 mM OA, 10 mM DA, or PBS. Forty-eight hours later the second PER-test was carried out, again using water, 0.1% (w/v) sucrose solution, and 43% (w/v) sucrose solution to elicit the PER.

In the first PER-test, the percentage of AMT-injected bees responding to the three stimuli was significantly lower than that of PBS-injected bees [**Figure 9B**; rm ANOVA, factor injection × sugar solution: *F*_2;1520_ = 6.368; *p* = 0.0012; Fisher LSD *post hoc* test for H_2_O: PBS vs. AMT: *p* < 0.001; for 0.1% (w/v) sucrose solution: PBS vs. AMT: *p* < 0.001; for 43% (w/v) sucrose solution: PBS vs. AMT: *p* > 0.001].

The effect of AMT was still observed 48 h after its injection (**Figure 9C**; rm ANOVA, factor injection: *F*_3;227_ = 5.7446; *p* = 0.0009, Fisher LSD *post hoc* test: AMT–PBS vs. PBS–PBS: *p* = 0.0212; **Figure 9D**; rm ANOVA, factor injection *F*_3;226_ = 4.924; *p* = 0.0025; Fisher LSD *post hoc* test: AMT–PBS vs. PBS–PBS: *p* = 0.0117). After a second injection with OA (AMT–OA) the PER-rate was no longer different from the control group that was injected with PBS at the same time point (PBS–PBS) (*p* = 0.2097). The PER rates of the groups AMT–OA and AMT–PBS differed significantly (*p* = 0.0005). The difference between the groups AMT–OA and PBS-OA was not significant (*p* = 0.9304) (**Figure 9C**).

A second injection with DA 24 h after the AMT injection (AMT–DA) did not increase the PER rate—there was still a significant difference between the groups AMT–DA and PBS–PBS (rm ANOVA, factor injection: *F*_3;226_ = 4.9244, Fisher LSD *post hoc* test: AMT–DA vs. PBS–PBS: *p* = 0.0011) and no significant difference between the groups AMT–DA and AMT–PBS (*p* = 0.4816) (**Figure 9D**).

Taken together, this experiment demonstrated that OA but not DA rescued the inhibiting effect of AMT on the percentage of hungry bees responding with a PER to water, 0.1% (w/v) and 43% (w/v) sucrose solution. Thus, we conclude that OA is involved in enhancing the PER and thus the feeding response of hungry bees.

## Discussion

### The Hemolymph Glucose Concentration Depends on the Bees’ Feeding State and Is Affected by Octopamine

Here we investigated a role for OA in the counter-regulatory response to a glucose deficit and therefore examined the glucose metabolism, survival, and feeding behavior of hungry and sated bees. We demonstrated that the glucose concentration of the bees’ hemolymph depends on the bees’ feeding state and that the hemolymph glucose concentration is modulated by OA.

We report that OA enhanced the glucose concentration in bees that were fed with 4 μl of 30% (w/v) sucrose solution but did not affect the hemolymph glucose concentration in hungry bees and bees that were fed to satiation. In hungry bees OA might not have enhanced the glucose concentration because glucose stores were nearly empty. In contrast, in sated bees, a ceiling effect might have been observed, because the hemolymph glucose concentration was as high as possible, and, therefore, no further enhancement following an OA injection was observed. In line with a possible ceiling effect, we demonstrated that the OA receptor antagonist epinastine inhibited the hemolymph glucose concentration in sated bees. We conclude from these data that OA enhances the hemolymph glucose concentration as long as carbohydrate stores were available.

Support for our conclusion comes from an earlier study in the fruit fly *Drosophila melanogaster*, demonstrating a reduced hemolymph concentration of glucose and trehalose in flies, mutant for the tyramine-β-hydroxylase (Tβh) gene (Tβh^nM18^) encoding Tβh, which converts tyramine to OA (Li et al., 2016). These mutants showed higher insulin release rates than control flies suggesting an increased storage of carbohydrates. In line with this observation, in the nematode *Caenorhabditis elegans* the biosynthesis of OA has been shown to be upregulated upon starvation by upregulation of the tβh-1 gene activity (Tao et al., 2016).

The mechanisms underlying an OA-dependent release of glucose into the honeybees’ hemolymph remain unknown. However, hints toward a possible mechanism come from a study by Blatt and Roces (2001). In honeybees, three main sugars are found in the hemolymph: trehalose, glucose, and fructose (Fell, 1990). Blatt and Roces (2001) demonstrated that with an increasing metabolic rate, the hemolymph concentration of glucose and fructose relative to trehalose increased, such that the overall hemolymph sugar levels remained unchanged. Blatt and Roces (2001) concluded that trehalose synthesis was not rapid enough to maintain stable trehalose concentrations at high metabolic rates, i.e., when demand became too great. They suggested that the decreasing trehalose concentration might result in a feedback signal to the proventriculus eliciting release of sucrose into the ventricle. In the ventricle sucrose is cleaved into glucose and fructose, and both sugars are released from the ventricle into the hemolymph (Blatt and Roces, 2001). We demonstrated that OA increased the hemolymph glucose concentration. Thus, OA might enhance the metabolic rate such that the trehalose concentration decreases leading to an increase of hemolymph glucose concentration.

### The Effect of Octopamine on the Honeybees’ Survival Is Restored by Feeding

We demonstrated that hungry honeybees, which received a systemic injection of OA after 18 h of fasting, survived for a shorter time period afterward (without food) than control bees having received a PBS-injection did. We concluded that OA activates available energy stores during food shortage at the expense of long-term survival. Indeed, when bees were fed once following the OA treatment, survival was restored, indicating that feeding, i.e., energy intake, compensated for the increase in the metabolic rate by OA. We found that the OA receptor antagonist epinastine prolonged survival of the bees fed once, supporting an involvement of OA receptors in regulating the bees’ metabolic rate and thus survival. Moreover, this result suggests that blockage of OA-receptors slows down the mobilization of available energy, such that the bees’ survival is prolonged. In line with our observation, tβh^nM18^ mutant flies died later from starvation than wild-type controls did (Scheiner et al., 2014; Li et al., 2016). Moreover, an ectopic release of OA during starvation reduces survival (Li et al., 2016). These findings in fruit flies again suggested that an increased OA-level mobilizes and empties energy stores, leading to an accelerated starvation (Scheiner et al., 2014; Li et al., 2016). In *C. elegans*, blocking the biosynthesis of OA (by means of RNAi against the tβh-1 gene activity) has been shown to lead to contrary results, i.e., a reduced survival rate after 3 days of fasting compared to wild-type worms (Tao et al., 2016). This reduced survival rate is rescued by application of OA (Tao et al., 2016). In line with our interpretation, Tao et al. (2016) hypothesized that OA mobilizes energy stores. However, in *C. elegans* mobilization of energy stores seems to enable long-term survival instead of reducing it as has been observed in *D. melanogaster* and *A. mellifera*. The reason for this discrepancy remains unclear. However, it might well be that these differences in OA-dependent long-term survival are due to differences in energy storage and energy metabolism in these three invertebrate species.

We demonstrated that the hemolymph glucose concentration is near zero in bees starved for 18 h. Furthermore, we demonstrated that OA reduced the survival rate of bees that were already starved for 18 h. Since we hypothesized that OA increases the metabolic rate in honeybees, the question remains which energy stores are activated after such a long starvation period. Wang et al. (2016) reported that thorax and abdomen glycogen and triglycerides are decreased 12 h after starvation in honeybees. Thus, it might well be that in our experiments OA triggered the depletion of glycogen and triglyceride stores when applied 12 h after feeding and that this mechanism led to a decreased survival of honeybees. Interestingly, in *C. elegans* it occurred that OA induces the expression of a lipase gene resulting in lipid mobilization (Tao et al., 2016). Furthermore, in the cockroach, *Blaberus discoidalis*, OA has been shown to be a potent activator of fat body glycogen phosphorylase, an enzyme that is needed to mobilize glucose from glycogen stores (Park and Keeley, 1998).

### Octopamine Is Involved in Regulating the Honeybees’ Feeding Behavior

In addition to a role for OA in regulating the hemolymph glucose concentration, we found an involvement of OA in regulating the bees’ feeding behavior. We demonstrated that the PER, which is a component of the bees’ feeding behavior, depends on the feeding state of honeybees and not on repeated stimulations of the antennae with sucrose solution, which theoretically could result in habituation. Moreover, we found that systemic application of OA does not affect the PER to different sucrose concentrations in sated and hungry bees. However, applying the OA-receptor antagonist epinastine did reduce the PER in hungry bees, suggesting that in hungry bees a ceiling effect is observed for OA, i.e., that the maximum of OA has been released in hungry bees already, such that additional OA does not affect behavior anymore. Indeed, when we inhibited the biosynthesis of OA and DA using AMT, the PER rate of hungry bees is reduced and can be rescued by the application of OA but not DA.

Several studies in *Drosophila* fruit flies and the blowfly *Phormia regina* have demonstrated that the hunger state affects the PER via a modulation of the sugar sensitivity (Moss and Dethier, 1983; Inagaki et al., 2012, 2014; Marella et al., 2012; Scheiner et al., 2014; Kain and Dahanukar, 2015; Yapici et al., 2016). Neuropeptide F and DA have been shown to be involved in PER by enhancing the responsiveness of taste sensory neurons (Inagaki et al., 2012, 2014; Marella et al., 2012). Our data indicated that OA modulates the PER as well. In line with this notion, an earlier study in honeybees demonstrated that depleting the nervous system of monoamines by the use of reserpine inhibited the PER, which was restored in reserpinized unresponsive bees by injection of OA (Braun and Bicker, 1992). In fruit flies, a reduced PER in starved tβh^nM18^ mutant flies has been demonstrated, which is rescued by feeding with OA (Scheiner et al., 2014).

Our data in honeybees and data of Scheiner et al. (2014) in fruit flies clearly indicate that OA modulates the PER depending on the insects’ feeding-state. However, the exact mechanism remains unclear. OA could act as a neurotransmitter and/or as a hormone when it is released during starvation.

### Is Octopamine Mediating the Stress–Response in Insects?

In mammals, starvation results in an activation of central and sympathetic catecholaminergic neurons, which regulate the release of glucose into the blood and modulate feeding behavior (Nonogaki, 2000; Ritter et al., 2001, 2011; Li et al., 2014; Morton et al., 2014; Verberne et al., 2014, 2016). Our results indicate that OA plays a role in regulating the honeybees’ energy state and behavior in response to starvation, supporting the hypothesis that OA is the functional homolog of adrenalin and noradrenalin.

Previous studies in honeybees have demonstrated a role of OA in the context of different physiological processes. In seminal studies on appetitive learning the activation of an octopaminergic Vummx1 neuron or the injection of OA into brain structures critically involved in insect olfactory learning, replaced the unconditioned stimulus, i.e., a sucrose solution (Hammer, 1993; Hammer and Menzel, 1998). Therefore, it has long been hypothesized that OA is the transmitter of the reward system in honeybees and other insects. Lately this hypothesis has seemed controversial, because in the fruit fly OA plays a role in formation of aversive memories as well (Wu et al., 2013), and short-term, but not long-term, memory formation depends on OA (Burke et al., 2012). Thus, the role of OA in learning and memory formation of insects, including the honeybee, remains unclear. Furthermore, OA modulates sensory processes, like vision, olfaction, and gustation (Braun and Bicker, 1992; Erber and Kloppenburg, 1995; Kloppenburg and Erber, 1995; Scheiner et al., 2002; Rein et al., 2013), locomotor and heart activity (Fussnecker et al., 2006; Bloch and Meshi, 2007), and the bees’ division of labor and dance communication (Schulz and Robinson, 1999; Wagener-Hulme et al., 1999; Barron et al., 2002, 2007; Schulz et al., 2002; Barron and Robinson, 2005; Giray et al., 2007; Lehmann et al., 2011; Reim and Scheiner, 2014).

Interestingly, noradrenalin and adrenalin modulate taste and olfaction, play a role in cardiovascular regulation and affect memory formation in mammals as well (Herness et al., 2002; Doucette et al., 2007; Roozendaal et al., 2008; Verberne et al., 2014; Tank and Lee Wong, 2015; Ness and Calabrese, 2016; Doyle and Meeks, 2017). Given that OA is a functional homolog of noradrenalin and adrenalin in regulating hunger-stress our results support the notion that OA has similar functions as these two catecholamines in triggering the animal’s physiological and behavioral stress–responses (Corbet, 1991; Roeder, 2005; Even et al., 2012). Conceptualizing OA as an insect stress hormone would explain why physiological processes as different as locomotion and learning and memory formation are modulated by OA. However, it would still be an open question how the role of OA in the regulation of the bee’s division of labor fits into this concept. Interestingly, it has been demonstrated that stressors like the loss of foragers, starvation, and diseases impact the division of labor, i.e., accelerate the onset of foraging (Schulz et al., 1998; Toth and Robinson, 2005; Higes et al., 2008; Goblirsch et al., 2013). At the same time, it has been shown that the brain OA-level is higher in foragers than in nurse bees (Harris and Woodring, 1992; Schulz et al., 2002; Lehman et al., 2006) and that OA enhances the likelihood to forage (Barron et al., 2002; Schulz et al., 2002; Barron and Robinson, 2005). Thus, an age-dependent increase of OA up to a critical threshold might result in the induction of foraging. OA released as a physiological response to stress might add up to the age-dependent OA-concentration such that the critical OA-threshold to induce foraging is reached earlier and precocious foraging can be observed.

## Author Contributions

CB designed experiments, acquired data, analyzed data, interpreted data, and critically revised the manuscript; OS designed experiments, acquired data, analyzed data; JG analyzed data and interpreted data; RZ and AÖ acquired and analyzed data; DE acquired funding, conceptualized and designed experiments, supervised study, interpreted data, and wrote the manuscript.

## Conflict of Interest Statement

The reviewer VM and handling Editor declared their shared affiliation, and the handling Editor states that the process nevertheless met the standards of a fair and objective review. The authors declare that the research was conducted in the absence of any commercial or financial relationships that could be construed as a potential conflict of interest.

## References

[B1] Barron A. B. Maleszka R. Vander Meer R. K. Robinson G. E. (2007). Octopamine modulates honey bee dance behavior. *Proc. Natl. Acad. Sci. U.S.A.* 104 1703–1707. 10.1073/pnas.0610506104 17237217 PMC1779631

[B2] Barron A. B. Robinson G. E. (2005). Selective modulation of task performance by octopamine in honey bee (*Apis mellifera*) division of labour. *J. Comp. Physiol. A Neuroethol. Sens. Neural Behav. Physiol.* 191 659–668. 10.1007/s00359-005-0619-7 15889261

[B3] Barron A. B. Schulz D. J. Robinson G. E. (2002). Octopamine modulates responsiveness to foraging-related stimuli in honey bees (*Apis mellifera*). *J. Comp. Physiol. A Neuroethol. Sens. Neural Behav. Physiol.* 188 603–610. 10.1007/s00359-002-0335-5 12355236

[B4] Beutler R. (1936). Über den Blutzucker der Biene. *Z. Vgl. Physiol.* 24 71–115. 10.1007/BF00340968

[B5] Blatt J. Roces F. (2001). Haemolymph sugar levels in foraging honeybees (*Apis mellifera* carnica): dependence on metabolic rate and in vivo measurement of maximal rates of trehalose synthesis. *J. Exp. Biol.* 204 2709–2716. 11533121 10.1242/jeb.204.15.2709

[B6] Bloch G. Meshi A. (2007). Influences of octopamine and juvenile hormone on locomotor behavior and period gene expression in the honeybee, *Apis mellifera*. *J. Comp. Physiol. A Neuroethol. Sens. Neural Behav. Physiol.* 193 181–199. 10.1007/s00359-006-0179-5 17082965

[B7] Braun G. Bicker G. (1992). Habituation of an appetitive reflex in the honeybee. *J. Neurophysiol.* 67 588–598. 1578245 10.1152/jn.1992.67.3.588

[B8] Burke C. J. Huetteroth W. Owald D. Perisse E. Krashes M. J. Das G. (2012). Layered reward signalling through octopamine and dopamine in Drosophila. *Nature* 492 433–437. 10.1038/nature11614 23103875 PMC3528794

[B9] Corbet S. A. (1991). A fresh look at the arousal syndrome of insects. *Adv. Insect Physiol.* 23 81–116. 10.1016/S0065-2806(08)60092-2

[B10] Doucette W. Milder J. Restrepo D. (2007). Adrenergic modulation of olfactory bulb circuitry affects odor discrimination. *Learn. Mem.* 14 539–547. 10.1101/lm.606407 17686948 PMC1951793

[B11] Doyle W. I. Meeks J. P. (2017). Heterogeneous effects of norepinephrine on spontaneous and stimulus-driven activity in the male accessory olfactory bulb. *J. Neurophysiol.* 117 1342–1351. 10.1152/jn.00871.2016 28053247 PMC5350266

[B12] Elliott R. H. Matthews V. B. Rudnicka C. Schlaich M. P. (2016). Is it time to think about the sodium glucose co-transporter 2 sympathetically? *Nephrology* 21 286–294. 10.1111/nep.12620 26369359

[B13] Erber J. Kloppenburg P. (1995). The modulatory effects of serotonin and octopamine in the visual system of the honey bee (*Apis mellifera* L.). *J. Comp. Physiol. A* 176 111–118. 10.1007/BF00197757

[B14] Even N. Devaud J. M. Barron A. B. (2012). General stress responses in the honey bee. *Insects* 3 1271–1298. 10.3390/insects3041271 26466739 PMC4553576

[B15] Fell R. D. (1990). The qualitative and quantitative analysis of insecthemolymphe sugars by high performance thin-layer chromatography. *Comp. Biochem. Physiol.* 95A 539–544. 10.1016/0300-9629(90)90735-B

[B16] Felsenberg J. Gehring K. B. Antemann V. Eisenhardt D. (2011). Behavioural pharmacology in classical conditioning of the proboscis extension response in honeybees (*Apis mellifera*). *J. Vis. Exp.* 2011 2282 10.3791/2282 PMC3182661 21304470

[B17] Fussnecker B. L. Smith B. H. Mustard J. A. (2006). Octopamine and tyramine influence the behavioral profile of locomotor activity in the honey bee (*Apis mellifera*). *J. Insect Physiol.* 52 1083–1092. 10.1016/j.jinsphys.2006.07.008 17028016 PMC1712669

[B18] Gallo V. P. Accordi F. Chimenti C. Civinini A. Crivellato E. (2016). Catecholaminergic system of invertebrates: comparative and evolutionary aspects in comparison with the octopaminergic system. *Int. Rev. Cell Mol. Biol.* 322 363–394. 10.1016/bs.ircmb.2015.12.006 26940523

[B19] Giray T. Galindo-Cardona A. Oskay D. (2007). Octopamine influences honey bee foraging preference. *J. Insect Physiol.* 53 691–698. 10.1016/j.jinsphys.2007.03.016 17574568 PMC4193539

[B20] Goblirsch M. Huang Z.-Y. Spivak M. (2013). Physiological and behavioral changes in honey bees (*Apis mellifera*) induced by *Nosema ceranae* infection. *PLoS ONE* 8:e58165 10.1371/journal.pone.0058165 PMC3590174 23483987

[B21] Hammer M. (1993). An identified neuron mediates the unconditioned stimulus in associative olfactory learning in honeybees. *Nature* 366 59–63. 10.1038/366059a0 24308080

[B22] Hammer M. Menzel R. (1998). Multiple sites of associative odor learning as revealed by local brain microinjections of octopamine in honeybees. *Learn. Mem.* 5 146–156. 10454379 PMC311245

[B23] Harris J. W. Woodring J. (1992). Effects of stress, age, season, and source colony on levels of octopamine, dopamine and serotonin in the honey bee (*Apis mellifera* L.) brain. *J. Insect Physiol.* 38 29–35. 10.1016/0022-1910(92)90019-A

[B24] Herness S. Zhao F. L. Kaya N. Lu S. G. Shen T. Sun X. D. (2002). Adrenergic signalling between rat taste receptor cells. *J. Physiol.* 543 601–614. 10.1113/jphysiol.2002.020438 12205193 PMC2290507

[B25] Higes M. Martín-Hernández R. Botías C. Bailón E. G. González-Porto A. V. Barrios L. (2008). How natural infection by *Nosema ceranae* causes honeybee colony collapse. *Environ. Microbiol.* 10 2659–2669. 10.1111/j.1462-2920.2008.01687.x 18647336

[B26] Hrassnigg N. Crailsheim K. (2005). Differences in drone and worker physiology in honeybees (*Apis mellifera*). *Apidologie* 36 255–277. 10.1051/apido:2005015

[B27] Ihle K. E. Baker N. A. Amdam G. V. (2014). Insulin-like peptide response to nutritional input in honey bee workers. *J. Insect Physiol.* 69 49–55. 10.1016/j.jinsphys.2014.05.026 24952326

[B28] Inagaki H. K. Ben-Tabou De-Leon S. Wong A. M. Jagadish S. Ishimoto H. Barnea G. (2012). Visualizing neuromodulation in vivo: TANGO-mapping of dopamine signaling reveals appetite control of sugar sensing. *Cell* 148 583–595. 10.1016/j.cell.2011.12.022 22304923 PMC3295637

[B29] Inagaki H. K. Panse K. M. Anderson D. J. (2014). Independent, reciprocal neuromodulatory control of sweet and bitter taste sensitivity during starvation in Drosophila. *Neuron* 84 806–820. 10.1016/j.neuron.2014.09.032 25451195 PMC4365050

[B30] Kain P. Dahanukar A. (2015). Secondary taste neurons that convey sweet taste and starvation in the Drosophila brain. *Neuron* 85 819–832. 10.1016/j.neuron.2015.01.005 25661186

[B31] Kloppenburg P. Erber J. (1995). The modulatory effects of serotonin and octopamine in the visual system of the honey bee (*Apis mellifera* L.). *J. Comp. Physiol. A* 176 119–129. 10.1007/BF00197758

[B32] Lehman H. K. Schulz D. J. Barron A. B. Wraight L. Hardison C. Whitney S. (2006). Division of labor in the honey bee (*Apis mellifera*): the role of tyramine beta-hydroxylase. *J. Exp. Biol.* 209 2774–2784. 10.1242/jeb.02296 16809468

[B33] Lehmann M. Gustav D. Galizia C. G. (2011). The early bee catches the flower - circadian rhythmicity influences learning performance in honey bees, *Apis mellifera*. *Behav. Ecol. Sociobiol.* 65 205–215. 10.1007/s00265-010-1026-9 21350590 PMC3022154

[B34] Li A. J. Wang Q. Dinh T. T. Powers B. R. Ritter S. (2014). Stimulation of feeding by three different glucose-sensing mechanisms requires hindbrain catecholamine neurons. *Am. J. Physiol. Regul. Integr. Comp. Physiol.* 306 R257–R264. 10.1152/ajpregu.00451.2013 24381177 PMC3921309

[B35] Li Y. Hoffmann J. Stephano F. Bruchhaus I. Fink C. Roeder T. (2016). Octopamine controls starvation resistance, life span and metabolic traits in Drosophila. *Sci. Rep.* 6:35359 10.1038/srep35359 PMC5069482 27759117

[B36] Marella S. Mann K. Scott K. (2012). Dopaminergic modulation of sucrose acceptance behavior in Drosophila. *Neuron* 73 941–950. 10.1016/j.neuron.2011.12.032 22405204 PMC3310174

[B37] Morton G. J. Meek T. H. Schwartz M. W. (2014). Neurobiology of food intake in health and disease. *Nat. Rev. Neurosci.* 15 367–378. 10.1038/nrn3745 24840801 PMC4076116

[B38] Moss C. F. Dethier V. G. (1983). Central nervous system regulation of finicky feeding by the blowfly. *Behav. Neurosci.* 97 541–548. 10.1037/0735-7044.97.4.541 6615630

[B39] Ness D. Calabrese P. (2016). Stress effects on multiple memory system interactions. *Neural Plast* 2016 20 10.1155/2016/4932128 PMC4807050 27034845

[B40] Nonogaki K. (2000). New insights into sympathetic regulation of glucose and fat metabolism. *Diabetologia* 43 533–549. 10.1007/s001250051341 10855527

[B41] Paoli P. P. Donley D. Stabler D. Saseendranath A. Nicolson S. W. Simpson S. J. (2014). Nutritional balance of essential amino acids and carbohydrates of the adult worker honeybee depends on age. *Amino Acids* 46 1449–1458. 10.1007/s00726-014-1706-2 24623119 PMC4021167

[B42] Park J. H. Keeley L. L. (1998). The effect of biogenic amines and their analogs on carbohydrate metabolism in the fat body of the cockroach *Blaberus discoidalis*. *Gen. Comp. Endocrinol.* 110 88–95. 10.1006/gcen.1997.7053 9514848

[B43] Rangel A. Camerer C. Montague P. R. (2008). A framework for studying the neurobiology of value-based decision making. *Nat. Rev. Neurosci.* 9 545–556. 10.1038/nrn2357 18545266 PMC4332708

[B44] Reim T. Scheiner R. (2014). Division of labour in honey bees: age- and task-related changes in the expression of octopamine receptor genes. *Insect Mol. Biol.* 23 833–841. 10.1111/imb.12130 25187440

[B45] Rein J. Mustard J. A. Strauch M. Smith B. H. Galizia C. G. (2013). Octopamine modulates activity of neural networks in the honey bee antennal lobe. *J. Comp. Physiol. A Neuroethol. Sens. Neural Behav. Physiol.* 199 947–962. 10.1007/s00359-013-0805-y 23681219 PMC3825135

[B46] Rether K. (2012). *Regulation of Haemolymph Glucose and its Importance in Sensory Sensitivity as Well as in Appetitive Learning and Memory in the Honeybee (Apis mellifera)*. Ph.D. dissertation, Universität des Saarlands Saarbrücken.

[B47] Ritter S. Bugarith K. Dinh T. T. (2001). Immunotoxic destruction of distinct catecholamine subgroups produces selective impairment of glucoregulatory responses and neuronal activation. *J. Comp. Neurol.* 432 197–216. 10.1002/cne.1097 11241386

[B48] Ritter S. Li A. J. Wang Q. Dinh T. T. (2011). Minireview: the value of looking backward: the essential role of the hindbrain in counterregulatory responses to glucose deficit. *Endocrinology* 152 4019–4032. 10.1210/en.2010-1458 21878511 PMC3444967

[B49] Roeder T. (2005). Tyramine and octopamine: ruling behavior and metabolism. *Annu. Rev. Entomol.* 50 447–477. 10.1146/annurev.ento.50.071803.130404 15355245

[B50] Roeder T. Degen J. Gewecke M. (1998). Epinastine, a highly specific antagonist of insect neuronal octopamine receptors. *Eur. J. Pharmacol.* 349 171–177. 10.1016/S0014-2999(98)00192-7 9671095

[B51] Roozendaal B. Barsegyan A. Lee S. (2008). Adrenal stress hormones, amygdala activation, and memory for emotionally arousing experiences. *Prog. Brain Res.* 167 79–97. 10.1016/S0079-6123(07)67006-X 18037008

[B52] Scheiner R. Pluckhahn S. Oney B. Blenau W. Erber J. (2002). Behavioural pharmacology of octopamine, tyramine and dopamine in honey bees. *Behav. Brain Res.* 136 545–553. 10.1016/S0166-4328(02)00205-X 12429417

[B53] Scheiner R. Steinbach A. Classen G. Strudthoff N. Scholz H. (2014). Octopamine indirectly affects proboscis extension response habituation in Drosophila melanogaster by controlling sucrose responsiveness. *J. Insect Physiol.* 69 107–117. 10.1016/j.jinsphys.2014.03.011 24819202

[B54] Schulz D. J. Barron A. B. Robinson G. E. (2002). A role for octopamine in honey bee division of labor. *Brain Behav. Evol.* 60 350–359. 10.1159/000067788 12563167

[B55] Schulz D. J. Huang Z.-Y. Robinson G. E. (1998). Effects of colony food shortage on behavioraldevelopment in honey bees. *Behav. Ecol. Sociobiol.* 42 295–303. 10.1007/s002650050442

[B56] Schulz D. J. Robinson G. E. (1999). Biogenic amines and division of labor in honey bee colonies: behaviorally related changes in the antennal lobes and age-related changes in the mushroom bodies. *J. Comp. Physiol. [A]* 184 481–488. 10.1007/s003590050348 10377981

[B57] Seoane-Collazo P. Ferno J. Gonzalez F. Dieguez C. Leis R. Nogueiras R. (2015). Hypothalamic-autonomic control of energy homeostasis. *Endocrine* 50 276–291. 10.1007/s12020-015-0658-y 26089260

[B58] Stevenson P. A. Hofmann H. A. Schoch K. Schildberger K. (2000). The fight and flight responses of crickets depleted of biogenic amines. *J. Neurobiol.* 43 107–120. 10.1002/(SICI)1097-4695(200005)43:2<107::AID-NEU1>3.0.CO;2-C 10770840

[B59] Tank A. W. Lee Wong D. (2015). Peripheral and central effects of circulating catecholamines. *Compr. Physiol.* 5 1–15. 10.1002/cphy.c140007 25589262

[B60] Tao J. Ma Y. C. Yang Z. S. Zou C. G. Zhang K. Q. (2016). Octopamine connects nutrient cues to lipid metabolism upon nutrient deprivation. *Sci. Adv.* 2:e1501372 10.1126/sciadv.1501372 PMC4928904 27386520

[B61] Toth A. L. Robinson G. E. (2005). Worker nutrition and division of labour in honeybees. *Anim. Behav.* 69 427–435.

[B62] Verberne A. J. Korim W. S. Sabetghadam A. Llewellyn-Smith I. J. (2016). Adrenaline: insights into its metabolic roles in hypoglycaemia and diabetes. *Br. J. Pharmacol.* 173 1425–1437. 10.1111/bph.13458 26896587 PMC4831313

[B63] Verberne A. J. Sabetghadam A. Korim W. S. (2014). Neural pathways that control the glucose counterregulatory response. *Front. Neurosci.* 8:38 10.3389/fnins.2014.00038 PMC3935387 24616659

[B64] Wagener-Hulme C. Kuehn J. C. Schulz D. J. Robinson G. E. (1999). Biogenic amines and division of labor in honey bee colonies. *J. Comp. Physiol. A Neuroethol. Sens. Neural Behav. Physiol.* 184 471–479. 10.1007/s003590050347 10377980

[B65] Wang Y. Campbell J. B. Kaftanoglu O. Page RE Jr Amdam G. V. Harrison J. F. (2016). Larval starvation improves metabolic response to adult starvation in honey bees (*Apis mellifera* L.). *J. Exp. Biol.* 219 960–968. 10.1242/jeb.136374 27030776

[B66] Wu C. L. Shih M. F. Lee P. T. Chiang A. S. (2013). An octopamine-mushroom body circuit modulates the formation of anesthesia-resistant memory in Drosophila. *Curr. Biol.* 23 2346–2354. 10.1016/j.cub.2013.09.056 24239122

[B67] Yang Z. Yu Y. Zhang V. Tian Y. Qi W. Wang L. (2015). Octopamine mediates starvation-induced hyperactivity in adult Drosophila. *Proc. Natl. Acad. Sci. U.S.A.* 112 5219–5224. 10.1073/pnas.1417838112 25848004 PMC4413307

[B68] Yapici N. Cohn R. Schusterreiter C. Ruta V. Vosshall L. B. (2016). A taste circuit that regulates ingestion by integrating food and hunger signals. *Cell* 165 715–729. 10.1016/j.cell.2016.02.061 27040496 PMC5544016

[B69] Yu Y. Huang R. Ye J. Zhang V. Wu C. Cheng G. (2016). Regulation of starvation-induced hyperactivity by insulin and glucagon signaling in adult Drosophila. *Elife* 5:e15693 10.7554/eLife.15693 PMC5042652 27612383

[B70] Zhang T. Branch A. Shen P. (2013). Octopamine-mediated circuit mechanism underlying controlled appetite for palatable food in Drosophila. *Proc. Natl. Acad. Sci. U.S.A.* 110 15431–15436. 10.1073/pnas.1308816110 24003139 PMC3780881

